# 16S rRNA metagenome analysis of gut bacteriome of Rohu (*Labeo rohita*) from Halda River and Kaptai Lake, Bangladesh

**DOI:** 10.1128/mra.00590-25

**Published:** 2025-09-08

**Authors:** Kazi Chamonara, Mohammad Sharif Uddin, Md. Habib Ullah Masum

**Affiliations:** 1 Department of Environmental Biotechnology, Faculty of Biotechnology and Genetic Engineering, Chattogram Veterinary and Animal Sciences University (CVASU) 130057, Chattogram, Bangladesh; 2 Department of Microbiology, Noakhali Science and Technology University (NSTU) 378872, Noakhali, Bangladesh; 3 Department of Genomics and Bioinformatics, Faculty of Biotechnology and Genetic Engineering, Chattogram Veterinary and Animal Sciences University (CVASU) 130057, Chattogram, Bangladesh; University of California Riverside, Riverside, California, USA

**Keywords:** Rohu fish, Halda River, Kaptai Lake, metagenome, gut microbiome

## Abstract

This research evaluated the gut microbiota of Rohu fish from the Halda River and Kaptai Lake in Bangladesh by 16S rRNA sequencing. Distinct microbial profiles were identified, with Halda samples concentrated in pathogens and Kaptai samples abundant in probiotics.

## ANNOUNCEMENT

Fish and fisheries are essential to Bangladesh’s economy, nutrition, and export potential. In 2020, Bangladesh ranked third globally in aquaculture, producing 1.25 million tons, which represents 11% of the world’s fish production (1). Fisheries contribute about 2.34% to the national GDP and 22.14% to agricultural GDP (2). Rohu (*Labeo rohita*), a key freshwater species, plays a significant role in aquaculture and food security and is commonly cultivated in ponds, floodplains, and rivers (3). The Halda River (HR) and Kaptai Lake (KL) are important fish production sites, known for the natural spawning of Indian major carps, such as Rohu (4, 5). Although gut microbiota is well-studied in many fish, research on Rohu’s microbiome remains limited despite its health importance (6–8). This research aimed to investigate the gut microbiome of Rohu fish using 16S rRNA gene amplicon sequencing.

Twelve mature Rohu fish were collected from March to April 2025 from two distinct aquatic areas in Bangladesh: HR and KL (Table 1). The fish samples were captured by local fishermen and promptly transported under chilled conditions (maintained at 4 °C) to the Central Genomics Laboratory, Department of Genomics and Bioinformatics, Chattogram Veterinary and Animal Sciences University, for subsequent processing. Sampling of intestinal contents (*n* = 12) was conducted within 12 h post-collection. From each fish, 100 mg of lower stomach content was extracted after scale removal. Genomic DNA was extracted using the commercially available DNeasy PowerSoil Pro Kit (Cat. no. 47014) according to the manufacturer’s instructions. The V1–V9 region of the 16S rRNA gene was amplified, and the amplicon went through end prep and dA tailing followed by barcode ligation using native barcoding kit (9). Adaptors were ligated using the NEBNext Quick Ligation Module. Libraries were pooled, mounted into a R10.4.1 flow cell, and sequenced using the MinION Mk1C. Data were obtained via MINKNOW v1.11.5.

**TABLE 1:** Summary of metadata and SRA accession numbers of the 16S rRNA amplicon sequences of Rohu gut samples, along with OTUs mapped against bacterial taxa, and raw, mapped unmapped reads.

Sample ID	Collection site	Coordinate	Source	Raw reads	Mapped reads (%)	Unmapped reads (%)	No. of observed OTUs	SRA accessions
HR1	Halda river, Hathazari	22.4997° N, 91.8717° E	River	23806	23077 (96.94)	729 (3.06)	91	SRR33766029
HR2	Halda river, Hathazari	22.4997° N, 91.8717° E	River	18462	17892 (96.91)	570 (3.09)	71	SRR33766028
HR3	Halda river, Hathazari	22.4997° N, 91.8717° E	River	10974	10648 (97.03)	326 (2.97)	52	SRR33766017
HR4	Halda river, Hathazari	22.4970° N, 91.8733° E	River	31423	30365 (96.63)	1058 (3.37)	123	SRR33766016
HR5	Halda river, Hathazari	22.4970° N, 91.8733° E	River	24259	23497 (96.86)	762 (3.14)	106	SRR33766015
HR7	Halda river, Hathazari	22.4970° N, 91.8733° E	River	16895	16443 (97.32)	452 (3.14)	85	SRR33766014
KL1	Kaptai lake, Rangamati	22.5978° N, 92.2133° E	Lake	41300	41180 (99.71)	120 (0.29)	89	SRR33766027
KL2	Kaptai lake, Rangamati	22.5978° N, 92.2133° E	Lake	24396	24339 (99.77)	57 (0.23)	108	SRR33766026
KL3	Kaptai lake, Rangamati	22.5978° N, 92.2133° E	Lake	37617	37562 (99.85)	55 (0.15)	117	SRR33766025
KL4	Kaptai lake, Rangamati	22.6447° N, 92.2019° E	Lake	32389	32347 (99.87)	42 (0.13)	71	SRR33766024
KL5	Kaptai lake, Rangamati	22.6447° N, 92.2019° E	Lake	11523	11362 (98.60)	161 (1.4)	78	SRR33766023
KL6	Kaptai lake, Rangamati	22.6447° N, 92.2019° E	Lake	30294	29887 (98.66)	407 (1.34)	190	SRR33766022

Data from the MinION (Mk1C) were processed using Guppy v6.3.2 in high accuracy mode, resulting in FASTQ reads with a quality score above 9. Adapters and barcodes underwent trimming using Porechop v0.2.4 (10), while low-quality bases were removed with Nanofilt (11). Selections were made for reads ranging from 1,300 to 1,600 base pairs, specifically for regions V1–V9. The taxonomic assignment was conducted using Epi2me’s wf-16s (Oxford Nanopore Technologies) (12) alongside the Standard-8 database (13) and Kraken2 (14). Default parameters were used except where otherwise noted.

**Fig 1:**
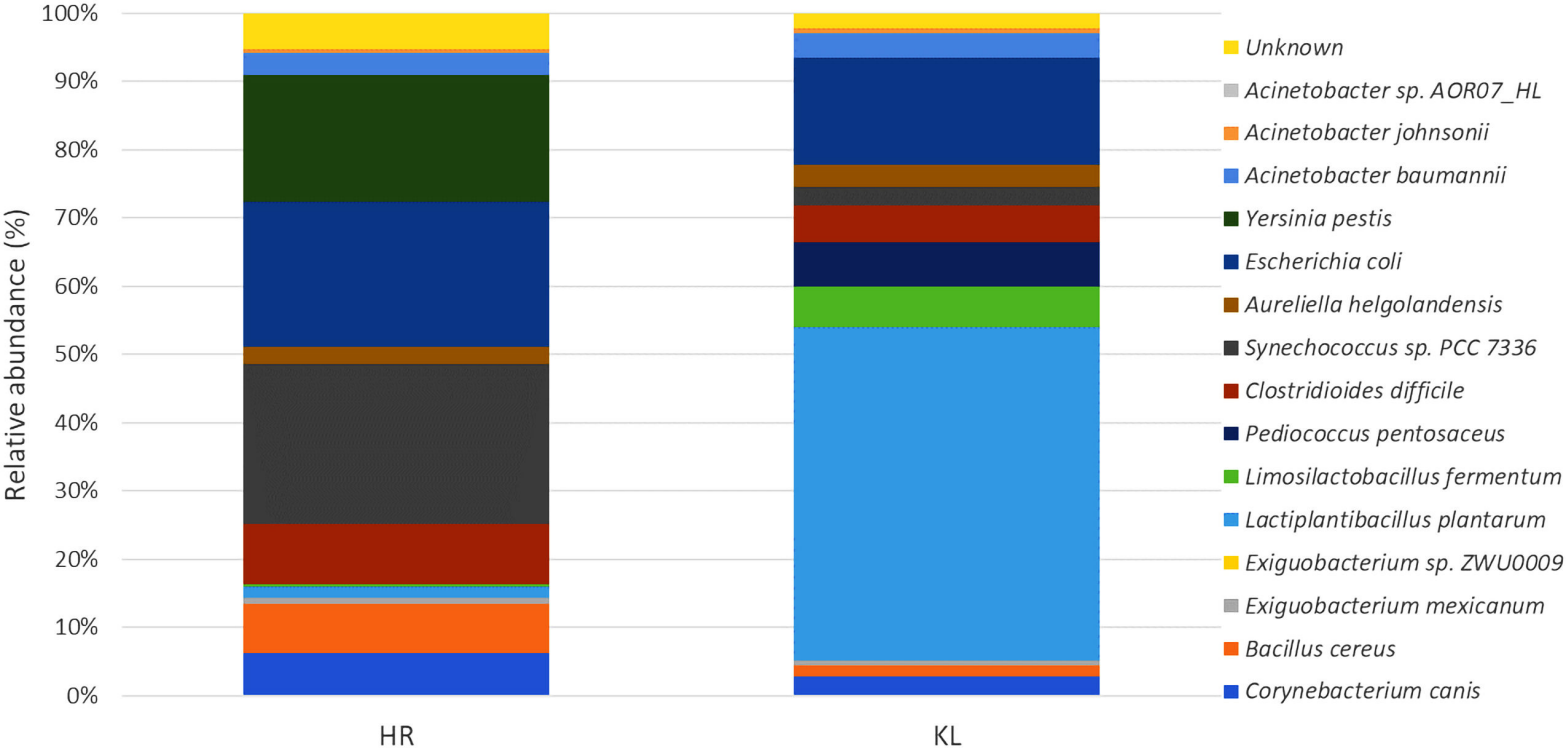
Relative abundances of the bacteriome in HR and KL samples at the phylum and species evels.

The 16S rRNA amplicon sequencing of 12 samples generated 303,338 raw reads (*N*_50_ = 1,300 kb), of which 298,599 (98.48%) quality reads mapped to 1,181 operational taxonomic units (OTUs) of Bacteria. Among these, 528 and 653 OTUs were identified in HR and KL fish gut samples, respectively, through the Standard-8 database (Table 1). In this study, the identified OTUs encompassed 5 bacterial phyla, 8 classes, 12 orders, 15 families, 16 genera, and species each. At the species level, *Synechococcus* sp. PCC 7336 (23.29%), *Escherichia coli* (21.28%), and *Yersinia pestis* (18.62%) were the most abundant in HR. In contrast, KL was primarily dominated by *Lactiplantibacillus plantarum* (48.80%), followed by *E. coli* (15.60%) and *Clostridioides difficile* (5.47%) (Fig. 1).

## Data Availability

All sequencing data generated in this study have been deposited in the NCBI BioProject database under accession number PRJNA1270017, and are publicly available through the Sequence Read Archive (SRA) (SRR33766029, SRR33766028, SRR33766017, SRR33766016, SRR33766015, SRR33766014, SRR33766027, SRR33766026, SRR33766025, SRR33766024, SRR33766023, SRR33766022).
